# Oral Bilateral Collagenous Fibroma: A previously unreported case and literature review

**DOI:** 10.4317/jced.54123

**Published:** 2018-01-01

**Authors:** Ana-Carolina Vasconcelos, Ana-Paula Gomes, Sandra Tarquinio, Eduardo Abduch-Rodrigues, Ricardo Mesquita, Karine Silva

**Affiliations:** 1 PhD, Post Graduate Program in Dentistry, School of Dentistry, Federal University of Pelotas, Pelotas-RS, Brazil; 2 MSc, Residence Program in Oral and Maxillofacial Surgery and Traumatology, Federal University of Pelotas-RS, Brazil; 3 PhD, Post Graduate Program in Dentistry, School of Dentistry, Federal University of Minas Gerais, Belo Horizonte-MG, Brazil; 4 MSc, Post Graduate Program in Dentistry, School of Dentistry, Federal University of Pelotas, Pelotas-RS, Brazil

## Abstract

Collagenous fibroma, also known as desmoplastic fibroblastoma, is a rare benign slow growing tumor particularly uncommon in the oral cavity. The aim of this study was to analyze the clinical and histopathological features of an oral collagenous fibroma as well as to compare this data with those reported in an English-literature review. The thirteenth case of collagenous fibroma in the oral cavity and the first to present clinically as a bilateral mass was described. A 48-years-old female patient was referred to a School of Dentistry, complaining about an asymptomatic swelling on the hard palate, lasting around ten years. The intraoral examination revealed two well-defined mass, bilaterally in the hard palate. An excisional biopsy was performed. Microscopically, the connective tissue consisted of dense collagen bundles in which were seen scarcely distributed spindle-shaped to stellate fibroblastic cells. Blood vessels were few, as well as inflammatory cells. Immunohistochemical staining was positive for vimentin, α-smooth muscle actin and factor XIIIa and negative for S-100, CD68, CD34, HHF35, desmin and AE1/AE3. The patient remains disease-free 24 months after excision. In conclusion, oral collagenous fibroma should be included in the differential diagnosis of bilateral sessile nodules in the oral cavity.

** Key words:**Connective tissue, mouth diseases, mouth neoplasms, oral diagnosis, oral pathology.

## Introduction

Collagenous fibroma (CF), also known as desmoplastic fibroblastoma, is a rare benign slow growing tumor, first described by Evans in 1995 (1). The pathogenesis of CF has not been completely established and it’s still unclear whether this lesion represents a reactive process or a true neoplasm (1,2). CF normally occurs in the skeletal muscle or subcutaneous tissue with a wide anatomic distribution, including arms, shoulders and feet (1,2).

The lesion, however, is particularly rare in the oral cavity (3-13). The intraoral cases have a propensity to occur in females in the fifth decade of life and usually present as a solitary, firm, well-circumscribed and asymptomatic mass (3). On histologic examination, CF is relatively paucicellular with spindle to stellate-shaped fibroblasts and myofibroblasts cells embedded in an abundant collagenous background. The fibroblasts are frequently binucleated or multinucleated. Poor vascularization and few inflammatory cells are also noted (1,13).

The treatment of choice for oral CF is total surgical excision, and neither local recurrence nor metastasis have been reported (5). Only few cases of CF affecting the intraoral region were reported in English-language literature (3-14). The aim of this article is to present the first case of bilateral CF in the oral cavity, discussing its clinical and histological characteristics with data from a literature review.

## Case Report

**Figure 1:**
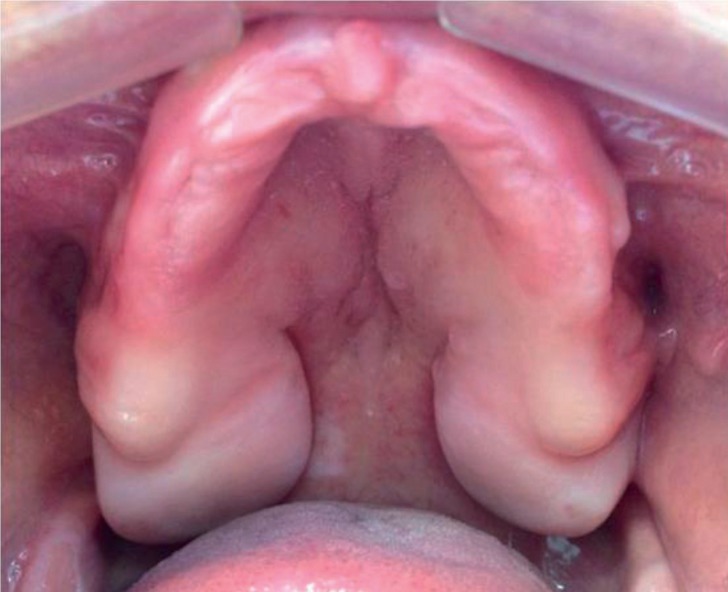
Intraoral view before surgery of the oral collagenous fibroma reported, revealing two well-defined sessile nodules, bilaterally in the hard palate, covered by a healthy mucosa.

**Figure 2:**
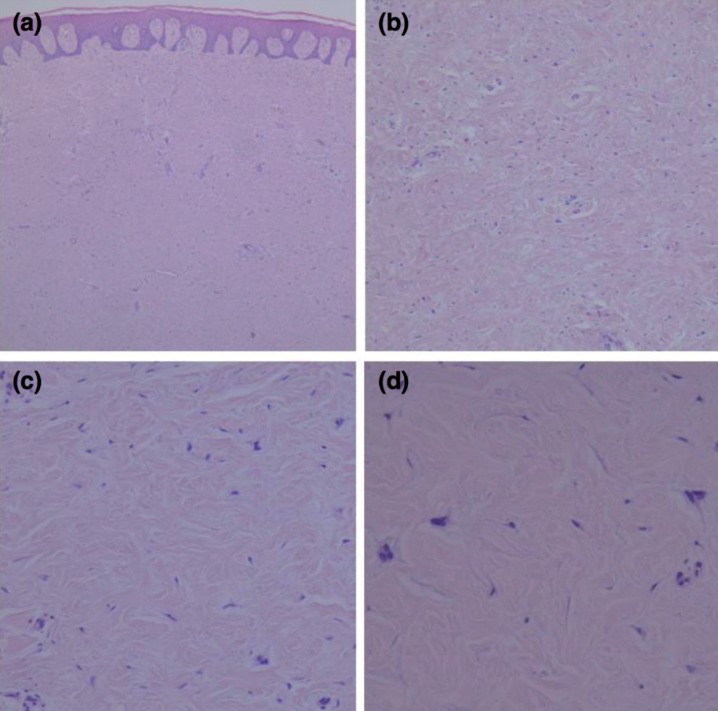
Photomicrographs of the oral collagenous fibroma reported (Hematoxylin and Eosin). (a) Normal epithelium coating a dense connective tissue (50X). (b) Dense collagen bundles with scarcely fibroblasts, poorly vascularization and few inflammatory cells (100X). (c) Spindle-shaped or stellate fibroblasts, some of them bi or trinucleated (200X). (d) Multinucleated cells embedded in a dense connective tissue (400X).

**Figure 3:**
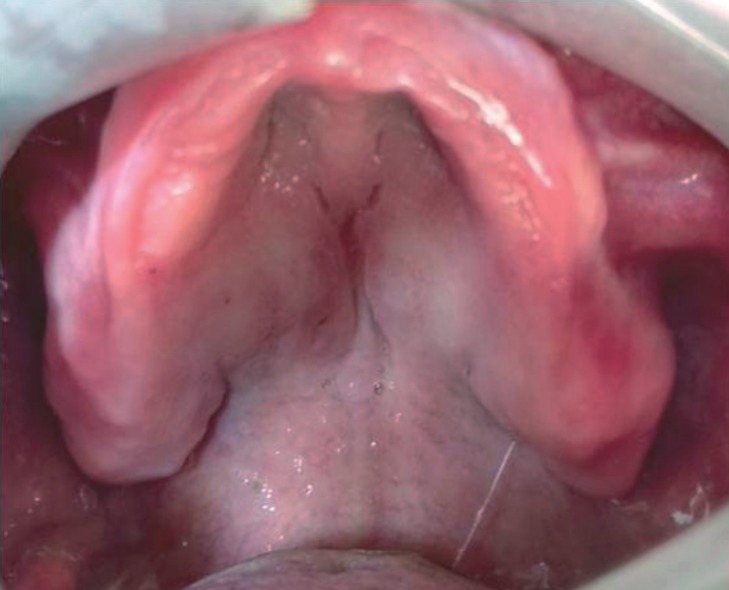
Intraoral view 24 months after surgery of the oral collagenous fibroma reported, displaying no clinical signs of recurrence.

A 48-years-old female patient was referred to the School of Dentistry, in August/2014, complaining about an asymptomatic swelling in the hard palate, lasting around 10 years. Medical, familial and dental histories were noncontributory. Intraoral examination revealed a well-defined submucosal sessile nodule, bilaterally, measuring each one 5.0 x 2.0 x 1.0 cm, with a firm consistency and covered by a healthy mucosa (Fig. 1). She was edentulous and used removable prosthesis for 30 years. No traumatic event or habit could be associated because the lesions were out of basal area of the denture. Extra oral inspection showed no abnormalities. An excisional biopsy was performed, in both lesions, considering the main clinical hypothesis of a benign mesenchymal tumor. Specimens were fixed with 10% formaldehyde and submitted for histopathological evaluation. Gross examination revealed two well-circumscribed, oval-shaped, fibroelastic masses, measuring 4.5 x 1.8 x 0.8 cm and 2.3 x 2.0 x 1.5 cm. Microscopically, it was observed a nonencapsulated, but well-delineated tumor covered by a normal oral epithelium. The connective tissue was dense, homogenous and poorly vascularized with few inflammatory cells. It was also observed dense collagen bundles with scarcely distributed spindle-shaped or stellate fibroblasts that were often multinucleated with large elongated to oval nuclei and delicate chromatin. No mitosis, necrosis, cystic degeneration, or infiltration of adipose tissue was noted (Fig. 2). Immunohistochemical staining was positive for vimentin, α-smooth muscle actin (α-SMA) and factor XIIIa and negative for S-100 protein, CD68, CD34, HHF35, desmin and anti-pan cytokeratin antibody (AE1/AE3). The patient showed no clinical signs of recurrence 24 months after surgical procedure (Fig. 3). The informed consent of the patient was obtained for this publication.

Literature Review

A review of the literature was done considering retrospective English written studies and cases located in oral soft tissues. The search was conducted in the PubMed and Web of Science Databases, using the following keywords: “oral collagenous fibroma” and “oral desmoplastic fibroblastoma”. Twelve articles met the inclusion criteria.

## Discussion

CF is a rare tumor in the oral cavity and, to date, no intraoral bilateral case of this lesion was reported (1,8). Considering the twelve cases of oral CF published in English-language literature ( Table 1), eight of them (75%) occurred in female, revealing a higher prevalence between women, as opposed to extraoral cases when the injury occurs mainly in males (1,3). In this same review, palate accounts for four cases (42%), buccal mucosa for two (16.7%), gum for four (33%) and tongue for one (8.3%). The mean age of these reported cases was 50.8 years, ranging from 8 to 87 years. The present case is in accordance with these main clinical features, occurring in a 48-years-old woman and located in the hard palate.

**Table 1:**
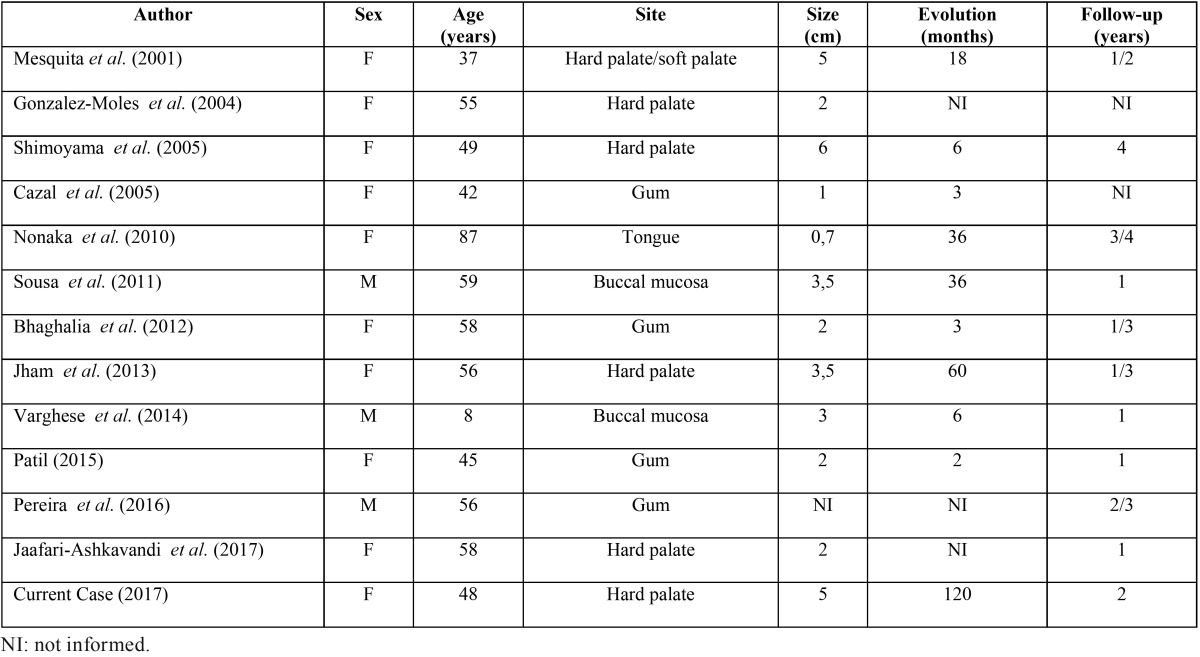
Clinical profiles of oral collagenous fibroma related in English-literature review and the current case.

Clinically, oral CF appears as slow growing, asymptomatic and well-circumscribed mass (3,5). The medium size of the intraoral CFs previously described is 2.8 cm (ranging from 0.7 to 3.5 cm) and all of them were solitary lesions which makes the current case a peculiar one. In the oral cavity, the differential diagnosis of CF should include neurofibroma, myofibroma, schwannoma and lipoma (2,3). Considering the bilateral location, clinical differential is more restrict and must include gingival fibromatosis. However, gingival fibromatosis normally occurs in association with other clinical disorders or syndromes, presence of teeth is believed to be necessary for its occurrence and recurrence is a frequent event (15).

Microscopically, the lesion is characterized by stellate or spindle cells with a round nucleous, small nucleoli and delicate chromatin. Multinucleated fibroblasts are occasionally observed. The cells are embebed in a prominent collagenous background with inconspicuous blood vessels. Mitotic figures are rare or absent, and necrosis is not observed. The presence of high hyalinized collagenous, few cellularity and scarce inflammatory infiltrate are contributors for the diagnosis of CF (1,13). The microscopic differential diagnosis includes giant cell fibroma (GCF), inflammatory fibrous hyperplasia (IFH) and traumatic fibroma (TF) (3,8). Notable presence of inflammatory infiltrate is more common in IFH, GCF and TF, as well as high cellularity, but rarely the cells are binucleated or stellate shaped (2,8). Moreover, gingival fibromatosis, a possible differential diagnosis, reveals major cellularity when compared with CF and it presents an epithelial surface with rete ridge activity which is not seen in CF (15).

The scarce inflammatory infiltrate seen in our case exclude the hypothesis of trauma, ruling out the diagnosis of IFH and even TF, like affirmed by Gonzalez-Moles *et al.* (4). In the present case, neither traumatic injury nor an inciting event could be identified, similarly to other reported cases (3,5,7). It is important to set that the use of removable prosthesis shows no relationship to the occurrence of the lesions because it is located out of basal area of the denture. In the present literature review there was no report of a tumor recurrence or metastasis after total surgical excision (3,5-13). Similarly, the present case was disease-free for 24 months.

The immunohistochemistry can be helpful to the differential diagnosis with other soft tissue lesions and is an important tool to make exclusion diagnosis, since there are many tumours with similar characteristics (6,13). The immunohistochemical profile of the 13 intraoral CF cases, including the present case, shows difuse positivity to vimentin in all ten performed cases, revealing the mesenchymal origin of the lesional cells (3,5-9,14). The imunnopositivity to α-SMA in seven of nine cases can explain a possible myofibroblastic origin of some cells (2,3,5-7). Our case is not reactive to HHF35 or Desmin. These antibodies were also not expressed or not performed in the majority of reported cases (3-11,14), making difficult the interpretation of the results. The negativity immunoprofile for S-100, CD34, CD68 e AE1/AE3 was observed when the technique was carried out (3,5,7,8,10,14), as well as in our case. This negativity can exclude lesions with neural, vascular or epithelial origin (9). The immunohistochemical profiles found in oral cases of CF revealed a marked positivity for vimentin and α-SMA when compared with others antibodies (3-14). This finding suggests that these lesions are associated with a mesenchymal origin with no neural or endothelial relationship.

In this way, although the histologic pattern is not exclusive of CF, the association between clinical, histopathological and immunohistochemical features strongly supports the diagnosis of this lesion for the described case.

The present work describes the thirteenth case of oral CF in English-language literature and the first to present clinically as a bilateral mass. This paper expands the spectrum of clinical manifestations of oral CF and demonstrates that this lesion should be included in the differential diagnosis of bilateral sessile nodules. Additionally, it is important to discuss the characteristics of this unusual pathology to better understand their clinical, pathological and immunohistochemical profile, helping clinicians and pathologists to conduct the correct diagnosis.
